# Ectoparasitism of the Flightless *Drosophila melanogaster* and *D. hydei* by the Mite *Blattisocius mali* (Acari: Blattisociidae)

**DOI:** 10.3390/insects14020146

**Published:** 2023-01-31

**Authors:** Katarzyna Michalska, Agnieszka Mrowińska, Marcin Studnicki

**Affiliations:** 1 Section of Applied Entomology, Department of Plant Protection, Institute of Horticulture Sciences, Warsaw University of Life Sciences, Nowoursynowska 159, 02-776 Warsaw, Poland; 2 Department of Biometry, Institute of Agriculture, Warsaw University of Life Sciences, Nowoursynowska 159, 02-776 Warsaw, Poland

**Keywords:** *Blattisocius*, *Drosophila*, predatory mite, phoresy, ectoparasite

## Abstract

**Simple Summary:**

There are a number of reports on the dispersal of predatory mites on insects, although much less is known about their mutual interactions and possible relationships. While some mite species use insects only as means of transport, others also feed on them, significantly lowering the carriers’ fitness. *Blattisocius mali* is a promising predatory mite for pest control, and a thorough understanding of its dispersal pathways in crops could be crucial for successful plant protection. So far, it has been reported as transported by several species of drosophilids. Our research indicates an ectoparasitic relationship between this species and drosophilid fruit flies. We used the flightless *D. melanogaster* and *D. hydei* commercially raised as live pet food. Female mites not only attached to flies but also fed on them, and their presence resulted in an increase in fly mortality. Although both fly species used similar defense tactics, mites had more difficulties getting onto *D. hydei*. Whether the wild *D. hydei* and *D. melanogaster* can also transport *B. mali* will be shown by further laboratory and field studies.

**Abstract:**

Predatory mites dispersing by means of insects are often ectoparasites and may use various tactics to get onto the host, counteract its defenses, and diminish its survival. *Blattisocius mali* is a promising biological control agent which has been reported as transported by several drosophilid species. Our goal was to determine the type of relationship between this mite and fruit flies. We used flightless females of *Drosophila melanogaster* and *D. hydei*, which were commercially raised as live pet food. The predatory females mostly attacked the tarsi of the flies and then preferentially moved to the cervix or close to coxa III, where they eventually drilled their chelicerae and started feeding. Although both fly species used similar defensive tactics, more *B. mali* females did not attack *D. hydei* or did so with a delay, and a higher percentage of mites fell off the *D. hydei* tarsi during the first hour of observation. After 24 h, we noted the increased mortality of flies exposed to the presence of mites. Our study indicates the ectoparasitic relationship of *B. mali* with drosophilids. However, further research is needed to confirm the transport of this mite on wild *D. hydei* and *D. melanogaster,* both in the laboratory and under natural conditions.

## 1. Introduction

Spreading using insects is a common mode of dispersal in mites [1,2,3,4,5]. Relationships between mites and the insects that carry them can take many forms, ranging from mutualism or commensalism to parasitism and predation [6,7]. As stated by Bartlow and Agosta [5], in the case of a phoresy one organism, the phoront transfers from one site to another with the aid of another organism, the dispersal host, and attaches to it, as a result of seeking or waiting for the host. As emphasized by other researchers, phoresy is temporary, and the phoront does not feed on the host or develop during transfer [1,4]. Predatory mites can also be transported by insects [4]. While some species are phoretic, such as e.g., *Poecilochirus carabi* G. Canestrini and R. Canestrini on the burying beetle *Nicrophorus vespilloides* Herbst [8] or *Parasitellus fucorum* (De Geer) on bumblebees *Bombus* sp. [9], others are ectoparasites that feed on insects during transport, such as *Macrocheles muscaedomesticae* (Scopoli) and *M. subbadius* (Berlese) [10,11]. Moreover, both phoronts and ectoparasites can also feed on eggs laid by the hosts and also in further developmental stages [8]. Despite many reports dealing with mite transfer by insects [5,12], there is a scarcity of detailed behavioral observations on the tactics employed by predatory mites to get onto the body of insects or the defense reactions of their carriers, which, apart from various ecological factors, could also determine the preference of predators towards the host species.

Over a dozen mite species are reported as associated with drosophilid flies, both in the wild and in laboratory cultures [13,14,15,16,17,18]. One of the predators on the list is *Blattisocius mali* (Oudemans). It is a promising biological control agent for acarid mites, eggs of the potato tuber moths, and nematodes [19,20,21,22,23,24,25]. However, there are still many gaps in our knowledge of its ecology and behavior, including the range of prey species and its dispersal methods.

*Blattisocius mali* belongs to Blattisociidae (Mesostigmata), a family of cosmopolitan predatory mites found both in the soil and in litter, on plants and stored products, and is frequently associated with rodents, insects, and birds [20]. Among species of *Blattisocius* Kegaan, *B. keegani* Fox, *B. dendriticus* (Berlese), *B. patagorium* Treat, and *B. tarsalis* (Berlese) have been reported as transported by noctuiid and pyralid moths [26,27,28,29]. Moreover, in *B. patogorium* and *B. tarsalis,* an ectoparasitic relationship with moths has been suggested [27,30]. Others, such as *B. apis* Basha and Yousef and *B. trigonae* Radhakrishnan and Ramaraju, were found on the body of honey bees, while *B. capsicum* Basha and Yousef was found in association with juveniles of psocopterous species inhabiting stored hot peppers [31,32].

So far, *B. mali* has been recorded only in fruit flies (Drosophilidae) from both the Drosophilinae and Steganinae subfamilies. In Finland, the mites (mostly deutonymphs and occasionally females) have been found on *Drosophila littoralis* Meigen, *D. montana* Stone, *D. ezoana* Takada and Okada, and *D. lummei* Hackman [15]. In the USA and Mexico, *B. mali* has been attached to *D. hexastigma* Patterson and Mainland [16]. Finally, in Hungary, Kerezsi et al. [17] found females of this mite on Steganinae fruit flies, *Phortica semivirgo* Máca. *Blattisocius mali* has also been reported as associated with drosophilids in laboratory cultures [15].

Phoretic mites attach themselves to insects in a variety of ways [1,4,5,33,34]. They can use chelicerae to grasp setae or folds of insect integument or attach to the host body by means of ambulaclar claws, while other mite phoronts possess special attachment devices, such as a pedicel formed by the secretion of anal glands in some Uropodina deutonymphs or the anal suckers of hypopus, which is a modified deutonymphal stage of some acarid mites. According to Lehtinen and Aspi [15], chelicerae tips of *B. mali* seemed to be inserted through the integument of fruit flies, which suggests that the association of this mite with drosophilids could be parasitic.

The aim of this study was to determine the type of relationship between *B. mali* and drosophilid flies. First, we examined the behavioral tactics the mite uses to get onto the body of the flies, where it preferentially attaches itself, and what the influence of the drosophilid species could be on this process. Secondly, we checked whether *B. mali* feeds on the fruit flies during transportation, and thirdly, whether the dispersal of the mite with the aid of the flies affects the survival of its carriers.

## 2. Materials and Methods

### 2.1. Insects and Mites

The stock population of *B. mali* and the mold mite *Tyrophagus putrecentiae* (Schrank) came from the laboratory mite cultures maintained in the Section of Applied Entomology, Department of Plant Protection at Warsaw University of Life Sciences (WULS). *Blattisocius mali* was identified by Dr. K. Michalska using the key by Karg [35] and subsequently by Prof. D. J. Gwiazdowicz. *Blattisocius mali* was mass-reared on various stages of *T. putrescentiae* in wheat bran on soaked foam platforms (22 cm × 15 cm × 2.5 cm), which were covered with foil and placed within broader vessels (50 cm × 70 cm × 20 cm) filled up with water. The cultures of *B. mali* were maintained in a climatic room, at 21–23 °C, with a photoperiod of 16/8 h (L/D). The population of *T. putrescentiae* was fed with instant yeasts and maintained in a desiccator (Chemland, Stargard, Poland) at 26 °C and 80–85% RH in darkness.

**Figure 1:**
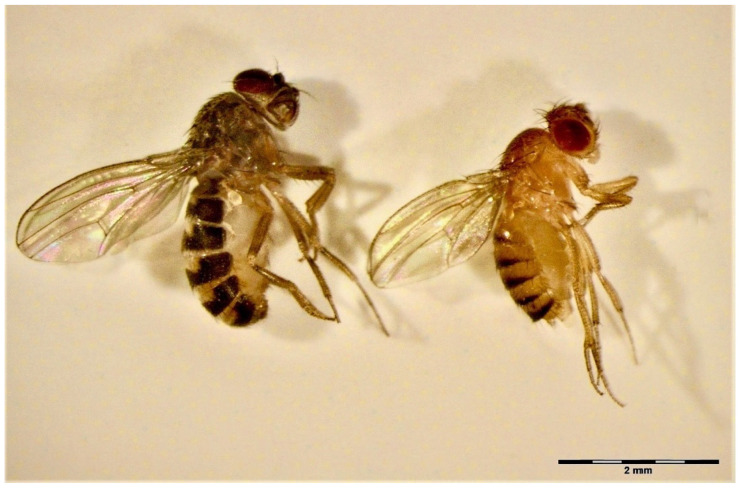
The flightless forms of *D. hydei* (**left**) and *D. melanogaster* (**right**) used in the experiments.

In this study, we used the flightless forms of two drosophilae species, *D. melanogaster* Meigen and *D. hydei* Sturtevant (Figure 1), both distributed commercially as live pet food [36]. The stock populations of these flies were obtained from the Amustela Zoological Centre, Warsaw, Poland. The mutation of various genes may lead to the flightlessness of drosophilids, from lack of wings or their deformation to dysfunction of the muscles controlling drosophilid flight [37,38,39,40,41]. The flightless fruit flies we used in this study had developed wings, but instead of flying, they were only hopping at a maximum height of several centimeters. We additionally identified them at the species level using DNA barcoding and the methods developed by Dabert et al. [42,43] and Mironov et al. [44]. Sequences generated in this study were deposited in GenBank (Supplementary File S1). The insects were reared on the standard fruit fly medium based on cornmeal, molasses, yeast, and propionic acid [45] in an incubator (Panasonic, Osaka, Japan) at 25 °C and a 12/12 (L/D) photoperiod.

We hypothesized that similar to wild drosophilids, flightless mutants can also transport *B. mali* on their bodies. Moreover, considering previous reports on the invasion of laboratory drosophilid cultures with *B. mali* [15], we hypothesized that this mite could feed on fruit flies and, as an ectoparasite, also suck up the body of adults. As the response of *B. mali* toward drosophilids could be innate, we tested naïve mites that had no previous contact with flies.

### 2.2. Experimental Set-Up

The tests were carried out within small observational chambers using pairs of randomly selected females of *B. mali* (*n* = 30) and females of *D. melanogaster* (*n* = 30) or *D. hydei* (*n* = 30). Apart from the treatment combination, a control was prepared in which female flies of *D. melanogaster* (*n* = 30) and *D. hydei* (*n* = 30) were kept singly, without mites. Observations were made at 5–50× magnification using a stereo microscope (Olympus. Tokyo, Japan) connected to a cooled light source. The observational chambers were constructed from colorless, transparent 200-μL Eppendorf pipette tips (FL Medical, Torreglia, Italy) that were 3 cm long. The chamber had a vent (ca. 0.5 mm in diam) at the narrower end, and at the broader end, it was plugged with a piece of cotton wrapped in gauze. First, single fruit fly females were introduced into the chamber, and then (after ca. 10 min when the flies became calm and the chamber became filled with their odor), the single 24-hour-starved females of *B. mali* were released into it. In contrast to fully satiated mites, the idiosoma of hungry mites was flat and brown. The mites were transferred with the aid of a fine brush, while the fruit flies were transferred using an aspirator (handmade). The mites were starved in isolation within a conical chamber of glass cages (4 cm × 3 cm × 0.3 cm) following the methods described by Robertson [46]. The conical chamber had two openings, an upper opening of 0.8 cm in diameter and the bottom one of 0.3 cm in diameter. The openings were sealed from above with a coverslip and at the bottom with permeable filter paper using warm paraffin. The cages containing the starving mites were put into a desiccator that was maintained in an incubator at 23 °C and 80–85% RH, with a 16/8 h (L/D) photoperiod. 

The experiment was divided into two parts. In the first part, which lasted one hour, detailed ethological observations and time measurements of the *B. mali* attachments to *D. melanogaster* and *D. hydei* were conducted. We regarded the place of location of the mite’s mouthparts on the fly’s body as a site of the mite’s attachment. For the description of the flies’ thoracic morphology, we used the nomenclature from Fabian et al. [47]. The pairs of fruit fly or mite legs were numbered I, II, III, and IV. During the first hour, we also examined whether *B. mali* females fed on fruit flies and whether the flies defended themselves against them. The mite’s feeding on the fly was judged by the degree of filling of its idiosoma. We arbitrarily assumed three degrees of the mite’s idiosoma filling: flat, partially filled, and full. The times of particular mite behaviors were measured using a stopwatch.

In order to examine whether *B. mali* may remain attached to a fly and feed over a longer period of time and how this may affect the viability of the fly and the mite itself, observations were continued for the next 23 h. After the completion of 1-h observations, the position of the mite on the fly’s body, the degree of the filling of its idiosoma, and the viability of the flies and the mite were noted, and then the observational chambers with the flies and *B. mali* females, as well as the control flies, were placed in an incubator with a temperature of 23° C and a 16/8 h (L/D) photoperiod. Additionally, we kept the relative humidity in the incubator at 60–70% RH, which, as investigations by Shaffer et al. [48] and Kamal et al. [49] have shown, is suitable for both the mite and the files. The examinations of insects and mites were repeated after 2, 3, 4, 5, and 24 h, each time transferring the observational chambers from the incubator to the laboratory under the stereo binocular microscope. The examinations of idiosoma filling were conducted on *B. mali* females that stayed with flies, which were either living or already dead, and could be either attached to or separated from the flies’ bodies.

### 2.3. Statistical Methods

The statistical analyses were performed using R 4.2.1 software [50]. In the analysis of differences in the times of the predator’s location and final attachment on the body of both fly species, a one or two-factor generalized linear model (GLM) was used with the assumption of gamma distribution. As the location phases were observed on the same *B. mali* individuals, the analysis took into account the structure of variance and covariance of AR1. An overall assessment of differences in observations expressed as percentages was made using the chi-square test for contingency tables, while pairwise comparisons between observations were made using the chi-square test of independence. Data were presented as mean ± standard error (SE).

## 3. Results

The behavior of *D. melanogaster* females showed markedly greater mobility than *D. hydei* in the first 10 min, even before the mite was placed into the chamber. These females very quickly moved from one end of the chamber to the other, seeking an exit. In the following minutes, *D. melanogaster* flies, both from the control and the treatment combination, were more often seen in the narrower end of the chamber, where there was a vent. When squeezing into the chamber tip, they curled their legs under them. *Drosophila hydei* females behaved differently. After one or two attempts to squeeze into the chamber tip, they moved towards the wider end, and when stopped, they remained in a standing position with straight legs.

Both *D. melanogaster* and *D. hydei* females responded to the mite attack and could make several different attempts to get rid of the mite. When *B. mali* attached the chelicerae to the tarsus of the fly, then the fly often curled her leg up under her or began to run. To get rid of the mite, she flicked with her tarsus and hit it against the walls of the chamber. Alternatively, she began by grooming and attempted to remove the mite using a leg of the same or one of the other pairs. Similarly, when *B. mali* stuck its chelicerae in the wing, thorax, or abdomen, then the flies tried to throw the mite off, rubbing the places where the mite had attached intensively with their legs I, II, or III. The attacked fly could also jump or roll over on her back, waving all her legs intensely.

During the first hour of observation, the percentage of successful attempts to throw the mite off the tarsus was significantly higher in *D. hydei* than in *D. melanogaster* (χ^2^ = 10.24, df = 1, *p* = 0.0013). In *D. melanogaster,* only one trial out of 37 attempts to remove *B. mali* was successful (2.7%), while in *D. hydei*, 8 out of 21 trials (39%) were completed with the removal of the mite. By contrast, the percentage of successful attempts to throw *B. mali* off other body parts did not differ significantly between fly species (χ^2^ = 0.53, df = 1, *p* = 0.4687). *Drosophila hydei* females managed to remove the mite 4 times out of 8 attempts (50%), while *D. melanogaster* only once in 6 attempts (16.67%).

### 3.1. The Phases of the Mite’s Location on the Fly’s Body

Observations carried out in the first hour allowed for the distinction of 3 main phases of *B. mali* getting onto *D. melanogaster* or *D. hydei* and its final attachment to their body. The first one was attack. During this phase, the mite most frequently attached to the tarsus of the fly leg, rapidly sticking its chelicerae into the first, second, or third segment of the tarsus. Only when the fly ‘calmed down’ (stopped and did not wave its legs) did the mite begin to climb up the fly’s body. Either (1) it moved along the more or less straight leg, onto the cervix (neck), thorax, or abdomen, or (2) if the fly had curled its leg under her, it moved from the tarsus or another part of the leg which was touching or a short distance from the fly’s body, directly onto the fly’s body. In the latter case, the predator stretched legs I or legs I and II in front of him and hooking them on the protruding part of the fly’s body, passed onto its cervix, thorax, or abdomen. (3) *B. mali* also got on drosophilids when body parts other than the tarsus were in contact with the wall of the observation chamber or the chamber plug at its wider end. In this case, the mites stuck their chelicerae into the thorax, abdomen, or wings and much more rarely into the head or mouth parts of the flies (Supplementary File S2).

*Blattisocius mali* attacked the flies predominantly when the insects stopped and became motionless or began by cleaning their legs. In *D. melanogaster*, only 2 out of 25 mite attacks happened when the fly was moving. In *D. hydei*, only one such attack (out of 26 observed cases) was noted (Supplementary File S2). Not infrequently, *B. mali* females remained stationary at one of the ends of the chamber (e.g., on the plug) or nearby, and when the fly stopped there, the attack began. The predators could attack the fly from the front (e.g., when they were attaching to the tarsus I) or from the back (e.g., when attaching to the tarsus III and II), or from any other side if it happened to be close to the contact point of other parts of the fly’s body with the wall of the chamber.

The passage of the mite from the site of attack to the site of the final attachment on the fly’s cervix, thorax, or abdomen was mostly very slow, as if the mite were ‘creeping’, and took place with or without stops. The final attachment was preceded by a characteristic drilling of the mouthparts into the integument of the insect. After inserting the chelicerae, predators raised the idiosoma upwards and began waving legs IV, III, and even II and turning the body sideways intensively. It was only after some time that the predators stopped waving their legs, their bodies settled on the flies ‘for good’, and they became motionless.

During the first hour of observation, all the tested female predators attempted to attack *D. melanogaster* females, while in the case of *D. hydei*, 23.33% of *B. mali* females showed no interest in the insects (χ^2^ = 5.8, df = 2, *p* = 0.0158). The mite attacked the *D. melanogaster* fly after, on average, 479.77 ± 80.31 s from the moment of release to the chamber, while attacking *D. hydei* after a significantly longer time, on average after 729.09 ± 113.21 s (GLM: χ^2^ = 9.091, df = 1, *p* = 0.0089). We observed, in total, 31 mite attacks (and first attempts to attach) on *D. melanogaster* and 32 mite attacks on *D. hydei*. However, in the case of *D. hydei,* the percentage of failed attempts to attach at the attack site was significantly higher, at 53.13%, while in the case of *D. melanogaster*, it was only 6.45% (χ^2^ = 14.14, df = 1, *p* = 0.0002). The mite failed to attach to the tarsus, rim of the wing, head, or tip of the abdomen of *D. hydei*, and it was usually thrown off these sites by a defending fly (13 attempts), much more rarely dropping off by itself (4 attempts). During the attacks on *D. melanogaster*, the mite attempted to attach to the tip of the abdomen. Once, it was thrown off by a fly, and once, it fell off by itself.

**Figure 2:**
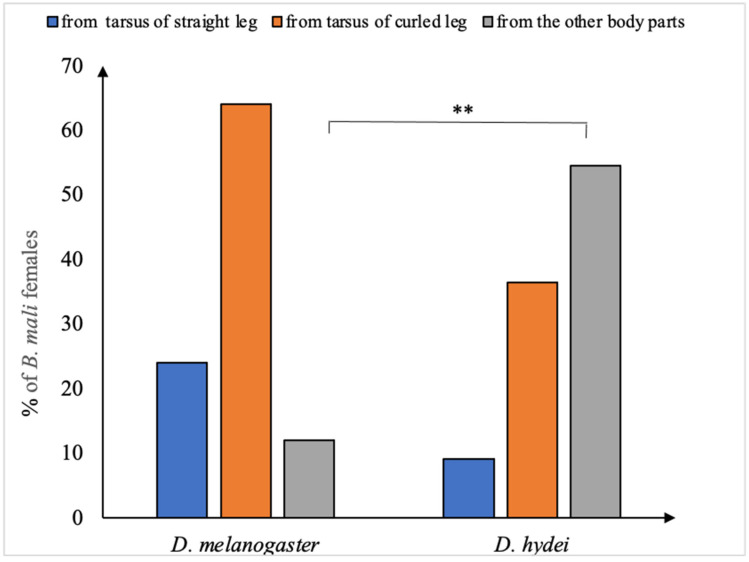
Percentage of *Blattisocius mali* females that located on *D. melanogaster* and *D. hydei* fruit flies during the first hour of observation using a particular way of getting on the fly’s body. ** 0.001 < *p* < 0.01.

Within the first hour of observation, there were significant differences in the percentage of mites located on the body of each fly species (χ^2^ = 11.73, df = 1, *p* = 0.0006). A total of 25 out of the 30 *B. mali* females kept with *D. melanogaster* (83.33%), and only 11 out of the 30 females (36.67%) kept with *D. hydei* got onto the cervix, thorax, or abdomen of the fly, where they finally drilled into its body. There were significant differences in the percentage of mites entering the body of *D. melanogaster,* either from the tarsus of a straight or curled leg or from the junction of the other fly’s body parts with the chamber wall (χ^2^ = 15.3, df = 1, *p* = 0.0004). Most *B. mali* females entered the body of *D. melanogaster* by moving from the tarsus, especially from that of the curled leg (Figure 2). By contrast, there were no significant differences in the percentage of *B. mali* females getting onto the body of *D. hydei* in either way (χ^2^ = 3.93, df = 1, *p* = 0.1398). However, in comparison with *D. melanogaster*, a much greater percentage of mites entered the *D. hydei* body by using the contact of body parts other than the fly’s tarsus with the chamber wall (χ^2^ = 9.07, df = 1, *p* = 0.0025) (Figure 2).

The total time it took for *B. mali* to finally attach to the fruit flies and also the time spent in each location phase varied greatly among mites (Table 1 and Supplementary File S2). The average total time needed for the final attachment to *D. melanogaster* was 1046.08 ± 175.35 s (300–4277 min-max; *n* = 25), and to *D. hydei* was 1193.46 ± 326.07 s (15–3105, min-max; *n* = 11) for *D. hydei*, and did not differ significantly between mites attacking either fly species (GLM: χ^2^ = 0.43749, df = 1, *p* = 0.5128). The fly species also had no effect on the average time the predator spent in a given phase of location on the fly’s body (GLM: χ^2^ = 0.23399, df = 1, *p* = 0.6296), and no significant interaction between the fly species and the location phase was detected (GLM: χ^2^ = 1.40291, df = 2, *p* = 0.2507) (Table 1). However, the effect of the phase type on the average time in a given phase was significant (GLM: χ^2^ = 7.39184, df = 2, *p* = 0.001). The mean time spent by a predator in the phase of attack and first attachment was significantly longer than that spent during the passage from the attack site to the site of final attachment (*p* ≤ 0.05) or during the drilling phase (*p* ≤ 0.05), both in *D. melanogaster* or *D. hydei* (Table 1). In neither fruit fly species, however, were there significant differences between the mean time of the two latter phases (*p* > 0.05).

### 3.2. Sites of Mite Attachment

The first hour observations showed that *B. mali* females moving from the attack site could have attached to flies in various places all over their body, but eventually, they began to drill with their chelicerae only in one of them. In *D. melanogaster,* there were 4 such sites on the cervix, thorax, and abdomen, while in *D. hydei,* there were 8 sites (Table 2).

In the following hours, we noted more cases of females that stuck in these sites as well as in other places on the flies’ bodies, although this time without a detailed examination as to whether their attachments were preceded by drilling or not. This includes the *B. mali* females that failed to attach during the first hour but became successful in the following hours. Some mites also changed the site of attachment. In total, over 24 h, we observed 5 sites of a mite’s attachments on the *D. melanogaster* cervix, thorax, and abdomen, while in the case of *D. hydei,* 12 of such sites were noted (Table 2).

**Figure 3:**
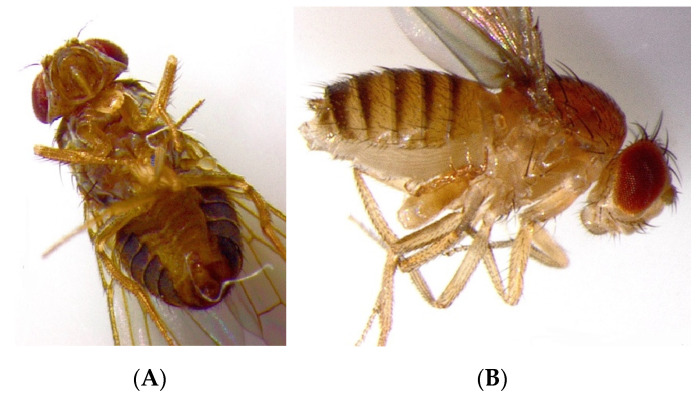
A female of *Blattisocius mali* attached to the cervix (neck) of a female of *Drosophila hydei* (**A**) and at the coxa III of a female of *D. melanogaster* (**B**).

The most frequently observed site of attachment of the *B. mali* chelicerae to the main fly body parts was the cervix area or sites close to the coxa III (Table 2) (Figure 3).

These sites were selected in the greatest percentage on the body of *D. melanogaster* both during the first hour (χ^2^ = 24.48, df = 3, *p* < 0.00001) and 24 h (χ^2^ = 46.06, df = 4, *p* < 0.00001). In *D. hydei*, however, the percentage of mites drilled into the cervix or near the coxa III during the first hour was similar to other places on the thorax or abdomen (χ^2^ = 3.2208, df = 7, *p* = 0.8639). Only for the total records of attachment, obtained during 24 h, were there significant differences in the frequency of *B. mali* females attached to the various sites on *D. hydei* (χ^2^ = 29.3, df = 11, *p* = 0.002), with a distinct predominance of females stuck, similarly as in the case of *D. melanogaster*, to the cervix and near the coxa III (Table 2).

The first-hour observations showed that the majority of mites that drilled into the cervix or at the coxa III reached these sites by first attacking the tarsi of the fruit fly’s legs (Table 3). Much more rarely, they attached to coxa III by climbing from other sites on the fly’s body. There were 2 out of 13 such mites on *D. melanogaster* that first attached to the wing or abdomen at the second sternite, and 1 out of 3 mites attacked the last abdominal tergite of *D. hydei* before reaching coxa III. Moreover, statistical analysis revealed that the percentage of *B. mali* females that drilled to the cervix (χ^2^ = 9.3, df = 2, *p* = 0.009562) or coxa III of *D. melanogaster* (χ^2^ = 8. 5128, df = 3, *p* = 0.03652) differed significantly depending on whether the mite attacked the tarsus I, II, or III or other sites of the fly’s body in contact with the observational chamber. The greatest percentage of mites reached the cervix of *D. melanogaster* by climbing from the tarsus I, while the greatest percentage of mites reached the fly’s coxa III by climbing from her tarsus III. A similar analysis was not performed for *D. hydei* due to insufficient sample size (Table 3).

As in the first hour of observation, in the following hours, some *B. mali* females were observed to be attached to the appendages of the fly body, i.e., the tarsus of a leg or to the wing, before reaching the cervix, thorax, or abdomen of the insects. Additionally, two mites on *D. melanogaster* and one on *D. hydei* did not reach the main body parts of flies but remained attached to the tarsus for 5 consecutive hours, and then after 24 h, were found outside the fly body or attached to the edge of its wing.

When the flies died, some of the *B. mali* females descended from them. Others, however, were stuck into the dead flies, and even during several hours of observation, changes in the sites of their attachment were not noticed.

### 3.3. Mite Feeding on Flies

**Figure 4:**
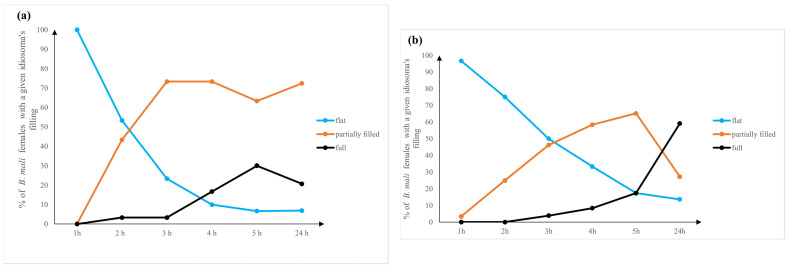
The degree of filling the idiosoma of *Blattisocius mali* females feeding on fruit flies of (**a**) *Drosophila melanogaster* and (**b**) *D. hydei* during 24 h observation.

Statistical analysis revealed the significant impact of time on the degree of filling up of *B. mali* idiosoma during a 24 h stay with either *D. melanogaster* (χ^2^ = 102.13, df = 10, *p* < 0.0001) or *D. hydei* (χ^2^ = 93.54, df = 10, *p* < 0.0001) (Figure 4a,b).

In the 24-h-starved mites, the body was flat and dark brown. They remained flat also when they became stuck to the tarsus of the leg or to the wing and failed to get onto a fly body. Two such cases have been observed on *D. melanogaster* and three cases on *D. hydei*. Only when *B. mali* females attached to the fly cervix, thorax, or abdomen did their idiosomas gradually fill. Initially, only a thickening of the back of the body, in the area of histerosoma, could be observed in the feeding mites. In the case of mites attached to *D. melanogaster*, a partial filling of the idiosoma was recorded in the second hour of observation. At this time, the idiosoma of one mite was already completely full. On *D. hydei*, the first cases of *B. mali* with completely filled idiosomas were not recorded until the third hour. The mites with full idiosomas were bulky, light brown on the dorsal side, and yellowish on the sides. Interestingly, after 24 h, most of the females feeding on *D. hydei* had completely filled idiosomas, while on *D. melanogaster*, partially filled idiosomas.

### 3.4. Effect of the Mite on Fly Survival

**Figure 5:**
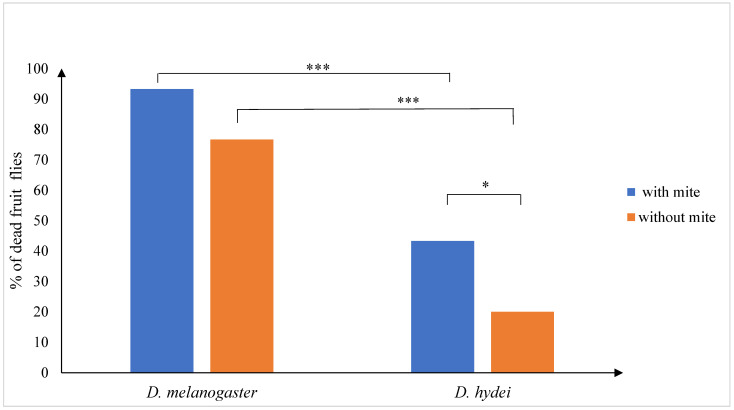
Mortality of *Drosophila melanogaster* and *D. hydei* female fruit flies during a 24 h stay with and without *Blattisocius mali* females in the observation chambers. Number of tested flies in each combination was *n* = 30. * 0.01 < *p* ≤ 0.05, *** *p* < 0.001.

During the 24 h experiments, significant mortality of fruit flies was observed. Statistical analysis revealed a significant effect of the fly species and the mite presence on fly survival (χ^2^ = 3.987, df = 1, *p* = 0.0354). The mortality was much higher in *D. melanogaster* than in *D. hydei,* both with *B. mali* (χ^2^ = 34.1, df = 1, *p* < 0.0001) and without the mite (χ^2^ = 27.8, df = 1, *p* < 0.0001), and no significant influence of the mite on survival of *D. melanogaster* was detected (χ^2^ = 2.2, df = 1, *p* = 0.0707) (Figure 5).

On the contrary, in *D. hydei*, after 24 h, in combination with a mite, a significantly higher percentage of dead flies of this species was recorded than in the control. (χ^2^ = 4.9, df = 1, *p* = 0.0443) (Figure 5).

## 4. Discussion

This study showed that *B. mali* females can not only be transported by females of the flightless forms of *D. melanogaster* and *D. hydei* but can also feed on them during transport and negatively affect their viability. This indicates an ectoparasitic relationship between this mite and the flies of the Drosophillidae family, as previously suggested by Lehtinen and Aspi [15], as well as Perez-Leanos et al. [16]. In our studies, both *D. melanogaster* and *D. hydei* showed defensive reactions. However, the species of fly had a significant impact on the effectiveness of the mite’s attack, as well as on its subsequent attachment to the fly’s body.

### 4.1. The Phases of the Mite’s Location on the Fly’s Body and the Fly’s Defense

In the observational chambers, hungry *B. mali* females took every opportunity to get onto the body of the flies and start feeding. This behavior was innate, as the tested mites had no previous experience with fruit flies or other insects. The whole process of embarkment on the host, however, could take *B. mali* females up to several dozen minutes, which might have been partly due to intense defense by the flies.

Undoubtedly, the morphology of the chelicera of *B. mali* females favored the capture of flies by the tarsus or wing. In their general plan, they are typical of most free-living mesostigmatic predatory mites, i.e., they are robust and chelate-dentate with retractable movable and fixed digits. In the Mesostigmata, these are used for a variety of purposes, including capture, penetration of the victim’s cuticle, and delivery of salivary enzymes that facilitate prey liquefaction [30,51]. The chelicerae of *B. mali* have relatively strongly curved ends, the movable digit has two teeth, and the fixed digit has one tooth [17,52]. They can act as pliers, clamping on the tarsus or the edge of the wing, indeed often, the clamp was so strong that the flies did not manage to throw them off the tarsus despite intensive waving and hitting the tarsus with the mite against the walls. This morphology of the chelicera probably also enabled them to hold on to the protrusions of the sclerites as they wandered through the fly’s body. However, while penetration of the cuticle in the case of small prey is a matter of a few seconds, in the case of flies, drilling into a much thicker cuticle took at least a few minutes and was accompanied by intense side-to-side body rotation and leg waving. Undoubtedly, the thick, serrate chelicera, curved at the end, make the piercing of insect integument difficult. For *B. mali*, there seems to be a kind of compromise between its polyphagy and the necessity of (facultative) transportation on an insect body. Other ectoparasites of drosophilid fruit flies with a similar structure of chelate-dentate chelicera may face this problem, such as *M. subbadius* [10] or *Proctolealeps regalis* DeLeon [53]. Piercing requires a significant modification of the mouthparts toward more slender and edentate digits, which is characteristic of some obligate mite parasites of invertebrates and vertebrates (e.g., dermanyssoid mites) [54,55]. It is also possible that selection pressures for a closer parasitic relationship between another representative of the genus *Blattisocius*, namely *B. patagorium* and noctuid moths, led to the development of an elongated edentate movable digit with a simultaneous reduction of the immobile digit. Individuals of this species not only fed on these insects but also mated, and the offspring could reach adulthood on the original host [27]. Interestingly, the larval movable digits of *B. patagiorium* are short and robust. However, as shown by Treat [27], the larvae can develop into protonymphs without food, and hence they probably do not require elongated and slender chelicerae, as the subsequent “feeding” stages of this mite do.

Our research showed that the species of fruit fly had a significant impact on the effectiveness of the mite’s attack, as well as on the way it entered and where it located on the body of the flies. Although both species of flies used similar tactics to throw off mites (flicking their legs while running and hitting the attached mite against the wall, rolling over on their back, rubbing the places with attached mites with their legs), the *D. hydei* flies were undoubtedly more ‘difficult’ hosts for *B. mali*. A greater percentage of female mites did not attack *D. hydei* at all, or their attack ended in failure. Perez-Leanos et al. [16] point to the possible preference of ectoparasites for certain species of Drosophilidae, due to the phylogeny of the host and its immunological, physiological, or biochemical characteristics. The predatory mite *M. subbadius* clearly preferred representatives of the subgenus *Drosophila* and repleta group (*D. hydei*) as opposed to the subgenus *Sophophora* (*D. simulans* Sturtevant), both in the field and in choice and no-choice experiments on immobilized (which were incapable of defending themselves) flies. It cannot be ruled out that *B. mali* may also have a preference for *D. melanogaster* in contrast to *D. hydei*. However, this requires further research. Undoubtedly, however, *D. hydei* defended itself more effectively while removing the mites from the tarsi. As a result, within the first hour of observation, more than 50% of the attacks ended in the failure of mite attachment to the tarsi, and of all *B. mali* females tested, only 1/3 finally entered the body and drilled their chelicerae into the abdomen, cervix, or thorax.

Drosophilid fruit flies’ defenses against ectoparasitic mites have been observed in *D. nigrospiracula* Patterson and Wheeler [56]. Their behavioral repertoire was wider than that of our flies. These were flying forms and were tested in much larger chambers. During an attack by *M. subbadius*, the flies flew up into the air, and they could also approach mites and appeared to exhibit reflex behavior in the form of sudden, brisk movements away from a mite. It should be mentioned that *D. hydei* is larger than *D. melanogaster* [57,58,59], and it may have greater vigor both in terms of mobility and the strength used during defense. Moreover, *D. hydei* could be more resistant to stress than *D. melanogaster*, which may also have resulted in its higher persistence in defense. In our study, the fruit flies were held in quite stressful conditions; apart from the exposure to the presence of mites, they were kept in a confined space, without the opportunity to escape and without food. Flying forms of *D. hydei* appear to have a higher tolerance to some kinds of stress, such as starvation and heat knockdown temperatures [60,61]. As shown by Homyk 1977 [37], some strains of flightless mutans of *D. melanogaster* may be more sensitive to stress in comparison to normal flies. Our 24 h observations appear to indicate differences in stress tolerance between the flightless forms of the two species. While the mortality of *D. hydei* in the control chamber (without mites) was 20%, in *D. melanogaster*, it was close to 80%.

Our study showed that *B. mali* females usually attempted to get onto flies when the insects stopped walking. Interestingly, they started to attack *D. hydei* after a longer time than *D. melanogaster*. It is likely that *D. hydei* flies were simply more mobile, and therefore, the mites had to wait longer for a convenient moment to attack. *Drosophila melanogaster* more frequently pressed into the narrow end of the chamber, where she also curled her legs. This behavior clearly favored the mites’ embarkment, as it was from the tarsus of a curled leg that *B. mali* females most frequently got onto the fly’s body. On the other hand, mites more frequently used the point of contact of the wall with the other fly’s body parts in order to enter *D. hydei* than *D. melanogaster.* One explanation for this could be that *D. hydei* flies are larger than *D. melanogaster,* and the contact of their bodies with the wall could have occurred more frequently. This, in turn, could be eagerly used by a mite. However, since the attacks directed at *D. hydei* tarsi often ended in defeat, *B. mali* females could seek ‘safer’ ways to enter the body of this fly species. This can be exemplified by the parasitoid *Trichogramma brassicae* Bezdenko, which is occasionally phoretic on white cabbage butterflies [62]. It avoided being kicked off, tending to climb onto butterflies’ wings instead.

### 4.2. Preferred Sites of Mite’s Attachment to the Fly’s Body

Mites transported by insects often show a preference for attachment sites on the host body. This preference may depend on the species and sex of the host [53]. It may also be associated with the selection of such places where the mite could avoid being removed by the host or mechanically damaged during transportation or where the host cuticle is soft and the mite’s mouthparts can easily be stuck into the host [63,64,65]. In our 24 h experiments, the fly’s cervix and the area close to coxa III were the most frequently selected sites of attachment by *B. mali* females. Indeed, these places have a flexible, intersegmental cuticle and were relatively safe, i.e., out of the reach of the tarsi of all three pairs of legs during the fly’s grooming.

The mite’s preference for the attachment to the fly’s cervix or at coxa III was already detected in the first hour of our experiment, but only with *D. melanogaster*. On *D. hydei*, the mite chose both the sites on the cervix and close to coxa III, as well as those on the thorax and abdomen. This was probably due to the fact that during the first hour, some *B. mali* females failed to attach to the *D. hydei* tarsus (from which they most often moved to the preferred places on the fruit flies) or even if they managed to do so, they had not started climbing the leg yet. Others got onto *D. hydei* from the contact of the chamber wall with other parts of the fly’s body and drilled themselves into less preferred sites on the fly’s thorax or abdomen. During the following hours, however, some mites finally reached the preferred places, which may have been the result of a gradual weakening of *D. hydei*’s defenses and/or the mites’ gain in experience. Interestingly, the sites which were preferred by *B. mali* in our study differ from those observed by Lehtinen and Aspi [15] and Kerezsi et al. [17]. It should be noted, however, that their specimens were collected from the field and referred to other fruit fly species. As suggested by Paraschive and Isaia [65], the location of mites on insects may be influenced by host population dynamics, diversity of mite species, time of season, or even the method of preserving hosts. In the studies by Lehtinen and Aspi [15], four Drosophilinae species were collected, and all *B. mali* specimens, mostly deutonymphs, were located under the fruit fly head, as in our study, but with the chelicerae stuck between the legs of the first pair, not in the cervix. Kerezsi et al. [17] reported only three *B. mali* females on *Ph. semivirgo*; two specimens on fly females were in a similar position as that described by Lehtinen and Aspi 1992 [15], and the third one, on a ventral part of the male fly thorax. In none of these studies were *B. mali* attached to the coxa III. Interestingly, both the cervix and coxa III were the preferred sites of *M. subbadius* in field studies on *D. nigrospiracula* and 13 other species of drosophilid fruit flies [16]. Our research showed that the choice of each of these sites by *B. mali* females depended on which *D. melanogaster* leg had been previously attacked. When mites attacked the tarsus I, they climbed the leg and most often attached themselves to the fly’s cervix. On the other hand, those attacking tarsus III located themselves at the coxa of these legs. Undoubtedly, this shortened the time of wandering about on the fly’s body and enabled the mite to get to each of these places as soon as possible.

### 4.3. Mite Feeding on Flies

As our study has shown, after drilling with their chelicerae into the body of flies, female mites, initially completely flat, gradually filled their idiosomas, most likely by sucking out hemolymph and other tissues dissolved in saliva. This feeding by predatory mites on the drosophilids carrying them was confirmed by Polak 1996 [10] using *M. subbadius* and radiolabeled *D. nigrospiracula*. Moreover, in the representative of the genus *Blattisocius*, *B. patagorium*, the behavior of the mite and scars on the body of some noctuid moth species suggested that this mite fed on its hosts during transportation [27]. Although in our study, we only registered a change in the idiosoma’s filling, the engorgement of *B. mali* females was similar to that noted during their feeding on eggs and first-stage larvae of *D. melanogaster* and *D. hydei,* as well as various stages of the mold mite *T. putrescentiae* (Michalska K., unpublished).

Only the *B. mali* individuals that attached to the tarsus or wing, as well as the individuals that had no interest in the fruit flies during the experiment, remained flat. This indicates that attack sites such as the fly’s tarsus or wing, while used by mites for attachment, are not suitable for feeding. This does not exclude, however, the possibility that the mite could feed on the tarsus of smaller prey that does not defend as vigorously as the fruit flies and has a much thinner cuticle as well. Examples are phytoseiid mites frequently attacking and then feeding on the tarsus and tibia of female spider mites [66].

Our study also suggests possible scavenging in *B. mali*. Several times, we observed that the mite, instead of getting off the freshly dead *D. hydei* or *D. melanogaster*, remained on the fly during subsequent hours with its chelicerae inserted in the same place on the insect body. Many species of mesostigmatic predators appear to be facultative scavengers, especially on freshly dead victims [51]. Scavenging has also been found in the genus *Blattisocius*, namely in *B. keegani* [29]. Interestingly, the addition of the freshly dead moth body of *Amyelois transitella* Walker to the moth’s eggs increased the predator’s fertility. In our study, even if a mite remained on the dead fly for many hours, we did not observe any changes in the filling up of its idiosoma. On the contrary, there was often a decrease in the filling up of the mite body. This may mean that the content of hemolymph in the dead flies gradually decreased, which, in turn, did not allow *B. mali* females full engorgement. Perhaps the mite could also feed a bit on a different type of the fly’s tissue through preoral digestion. This, however, requires further detailed research.

### 4.4. Effect of the Mite on the Fly’s Survival

Our experiments revealed the significant effect of the presence of *B. mali* on the viability of female fruit flies. In *D. hydei*, after 24 h in the chambers with a mite, the mortality of females was twice as high as that in the combination without a mite. In *D. melanogaster*, a similar analysis was not possible due to the high mortality of flies already in the first hour of the test, both in combination with a mite and in the control group. The increased mortality of *D. hydei* could have resulted not only from *B. mali* feeding but also from energy expenditure incurred by the flies during defense against a mite and during attempts to remove it or due to stress (lack of food, lack of opportunity to escape, etc.). As the experiments by Luong et al. [67] showed, the mere presence of *M. muscadomesticae* (Scopoli) mites, without their attachment to a *D. hydei* body, can significantly increase the energy expenditure of the fly, as manifested by its elevated CO_2_ production. A similar effect was found in *D. nigrospiracula* exposed to the presence of *M. subbadius* [68]. The negative impact of mite ectoparasitism on the fitness of the insects that carry them is also evidenced by the studies of other authors. According to Jalil and Rodriquez [69], the parasitism of the *M. muscaedomesticae* mite significantly reduced the survival rate of the housefly. In turn, the presence of *M. subbadius* on the body of *D. hydei* and *D. nigrospiracula* significantly shortened the duration of their flight, limiting the possibility of their dispersal [58]. The mite not only hinders the mating of *D. nigrospiracula* but also reduces the fertility of the females and males of this fly [10,70].

In summary, our research has shown that *B. mali* can act as an ectoparasite in relation to drosophilids. Not only can it feed on them during transport, but it probably also affects their survival and feeds on dead fruit flies. However, since we used the flightless forms of *D. melanogaster* and *D. hydei*, further research is needed on the wild forms of these species, both in the laboratory and in the field.

## Figures and Tables

**Table 1:** Time (mean ± SE, minimum and maximum, *n*-number of replications) of phases of *Blattisocius mali* location on the body of *Drosophila melanogaster* and *D. hydei* female fruit flies during the first hour of observation. a and b in raw indicate the significant differences between means (*p* ≤ 0.05).

Fruit Fly Species	Phases of the Mite’s Location on the Fly’s Body		
	1. Attack and First Attachment	2. Passage from the Attack Site to the Site of Final Attachment	3. Drilling into Fly Integument and Final Attachment
*D. melanogaster*	509.98 ± 86.52 **a** (157–2187; *n* = 25)	249.86 ± 113.50 **b** (20–2768; *n* = 25)	233 ± 81.51 **b** (7–2039; *n* = 25)
*D. hydei*	849.55 ± 269.70 **a** (2–2077; *n* = 11)	83.27 ± 27.07 **b** (0–237; *n* = 11)	269.27 ± 189.42 **b** (13–2143; *n* = 11)

**Table 2:** Sites of *Blattisocius mali* attachment to the cervix, thorax, or abdomen of *Drosophila melanogaster* and *D. hydei* female fruit flies noted during 24 h observations. For the first hour of observation, only the final attachments (that ended up with drilling) are given.

Fruit Fly Species	Site of Attachment	No of Predators Attached	
		1st h	24 h
*D. melanogaster*	cervix, ventral site	10	13
	thorax, dorsal site; at wing joint		1
	thorax, at the coxa II	1	1
	thorax, at the coxa III	13	17
	abdomen, dorsal site—at 5th tergite	1	1
*D. hydei*	cervix, ventral site	2	5
	cervix, lateral site	1	1
	thorax, ventral site, between proepisternum and profurcasternum	1	1
	thorax, dorsal site; at wing joint		1
	junction between thorax and abdomen; dorsal site	1	2
	thorax, at the coxa II	1	2
	thorax, at the coxa III	3	9
	abdomen, ventral site, at 2nd sternite	1	1
	abdomen, ventral site, at last sternite		1
	abdomen, dorsal site, at 5th tergite		2
	abdomen, dorsal site, at 6th tergite		1
	abdomen, dorsal site, at last tergite	1	2

**Table 3:** Number of *Blattisocius mali* females that drilled their chelicerae into the cervix, at coxa III or other sites on the thorax or abdomen of *D. melanogaster* and *D. hydei* according to the site of mite attack.

Site of Attack	Site of Drilling and Final Attachment of Chelicerae during 1st Hour of Observation					
	*D. melanogaster*			*D. hydei*		
	Cervix	Coxa III	Other Sites	Cervix	Coxa III	Other Sites
tarsus I	7	1		3	1	
tarsus II	1	3	1		1	1
tarsus III	2	7				
other body parts		2	1		1	4

## Data Availability

A description of the DNA barcoding identification of the studied flightless fruit flies to species level and some details on the behavior of *Blattisocius mali* and flightless *Drosophila melanogaster* and *D. hydei* are available as Supplementary Materials for download. Other data used in this study are available by email request to the corresponding author.

## Supplementary Materials

The following supporting information can be downloaded at: https://www.mdpi.com/article/10.3390/insects14020146/s1. File S1: DNA barcoding identification of the studied flightless fruit flies to species level; File S2: Some details on the behavior of *Blattisocius mali* and flightless *Drosophila melanogaster* and *D. hydei.* References [42,43,44] are cited in Supplementary Materials.
