# Supplementary material for: Ectoparasitism of the Flightless Drosophila melanogaster and D. hydei by the Mite Blattisocius mali (Acari: Blattisociidae)
Source: Insects. 2023 Jan 31;14(2):146. doi: 10.3390/insects14020146 (PMC9961106; doi:10.3390/insects14020146)
Supplement: Supplementary file 1 [file insects-14-00146-s001.zip › File S1-DNA barcoding identification of the studied flightless fruit flies to species level.pdf]

File S1: DNA barcoding identification of the studied flightless fruit flies to species level

Methods

DNA extraction, amplification, and sequencing

Flies were subjected to DNA extraction individually. After 48-72 h incubation in the ATL lysis buffer with 0.2 mg/ml Proteinase K (Qiagen, Hilden, Germany), total genomic DNA was purified from the lysate using the silica-column method. The mitochondrial (mt) cytochrome c oxidase subunit I (COI) gene fragment encompassing the DNA barcode (https://boldsystems.org/) was amplified using the primer set bcdF01 (CATTTTCHACTAAYCATAARGATATTGG) and bcdR04 (TATAAACYTCDGGATGNCCAAAAAA)[42]. A fragment of 18S rRNA gene was amplified using 18Sfw (CTTGTCTCAAAGATTAAGCCATGCA) and rev960 (GACGGTCCAAGAATTTCAC) primers [42]. A fragment of 28S rRNA gene was amplified using 28SF0001 (ACCCVCYNAATTTAAGCATAT) and 28SR0990 (CCTTGGTCCGTGTTTCAAGAC) primers [44]. PCRs, sequencing and sequence analyses were carried out as previously described [43].

The COI sequences were compared to GenBank nucleotide collection (nr/nt) using blast suite and Megablast algorithm to determine tick species. Generated sequences were deposited in GenBank under accession nos.: SUB12432515 KM001_bcdF01_C06_2022-11-16_B OQ058862-71(COI), SUB12432544 KM002_18SF01 OQ058966-68 (18S), and SUB12432564 KM002_28SF001 OQ058969-71 (28S).

Results

We identified COI sequences for 10 flies, five each representing both species. All the COI sequences enabled us to classify the analysed flies into *D. melanogaster* and *D. hydei* with nearly 100% sequence identity. Drosophila melanogaster flies shared the same COI haplotype, while two haplotypes, differing in two nucleotide positions, were found among *D. hydei*. Sequences coding both 18S and 28S rRNA gene fragments confirmed the species identified using COI gene barcode showing 100% identities with reference sequences found in *D. melanogaster* and *D. hydei*.

References

42 Dabert, M.; Witalinski, W.; Kazmierski, A.; Olszanowski, Z.; Dabert, J. Molecular phylogeny of acariform mites (Acari, Arachnida): Strong conflict between phylogenetic signal and long-branch attraction artifacts. *Mol. Phylogenet. Evol.* **2010**, *56*, 222–241. https://doi.org/10.1016/j.ympev.2009.12.020.

43 Dabert, J.; Mironov, S.V.; Dabert, M. The explosive radiation, intense host-shifts and long-term failure to speciate in the evolutionary history of the feather mite genus *Analges* (Acariformes: Analgidae) from European passerines. *Zool. J. Linn. Soc.* **2022**, *195*, 673–694. https://doi.org/10.1093/zoolinnean/zlab057.

44 Mironov, S.V.; Dabert, J.; Dabert, M. A new feather mite species of the genus Proctophyllodes Robin, 1877 (Astigmata: Proctophyllodidae) from the Long-tailed Tit Aegithalos caudatus (Passeriformes: Aegithalidae)—Morphological description with DNA barcode data. *Zootaxa* **2012**, *3253*, 54–61. https://doi.org/10.11646/zootaxa.3253.1.2.
