# Supplementary material for: Ectoparasitism of the Flightless Drosophila melanogaster and D. hydei by the Mite Blattisocius mali (Acari: Blattisociidae)
Source: Insects. 2023 Jan 31;14(2):146. doi: 10.3390/insects14020146 (PMC9961106; doi:10.3390/insects14020146)
Supplement: Supplementary file 1 [file insects-14-00146-s001.zip › File S2.pdf]

File S2: Some details on the behaviour of *B. mali* and flightless *D. melanogaster* and *D. hydei*

Blattisocius mali attacks of the fruit flies

Mite attacks on the head and mouthparts of the fruit flies were observed only in combination with *D. hydei*. One *B. mali* female attempted to embark onto the fly by jumping from the wall of the observational chamber directly onto the insect's eye. The *D. hydei* female's response was immediate. The fly jumped away vigorously, and at the same time the mite dropped off. This mite also attacked a female jumping from the chamber plug onto the vertex of the fly head. The fly instantly began grooming the head with legs I and removed the mite after 58 seconds. Another *B. mali* female stuck into the proboscis of *D. hydei* while the fly examined the substrate with it. After the mite's attack, the fly began to rapidly and alternately pull out and retract the proboscis. During one of the retraction movements, the mite got onto the fly's body and then stuck the chelicerae into its cervix.

Mite attacks on walking females were noted very rarely. In D. hydei, this was observed only once. The fly was turning back at the end of the vial, then the mite attached close to the last tergite of the fly abdomen. In the case of D. melanogaster, a female once walked slowly along the chamber and the mite ran up and stuck its chelicerae into the tarsus II, and the second time, when the fly was turning back at the end of the chamber, the mite attacked tarsus I.

Time of phases of *Blattisocius mali* location on the body of fruit flies during the first hour of observation

Although generally the attack and first attachment phase lasted from several to several dozen minutes, we observed two B. mali females that needed only a few seconds to get from the chamber wall onto a D. hydei abdomen, and immediately after landing they began to drill, omitting the passage phase. It usually took B. mali a few minutes to drill into the fly integument and become motionless. However, two cases were observed in which the predators attached to the coxa II of D. melanogaster and D. hydei, and the time of drilling in these sites was exceptionally long, over half an hour.

Attachment of *B. mali* to dead fruit flies

Six mites were observed attached to dead D. melanogaster (5 mites attached at coxa III and 1 mite at the cervix) and 2 mites to dead D. hydei (one B. mali female was attached at the junction between thorax and abdomen on the dorsal site, and another one at the coxa II).

Feeding of *B. mali* when attached to the tarsus of fruit flies

One mite was observed attached to the fly's tarsus after the 1st, 2nd, 3rd, 4th, 5th and after 24 hours, another was observed on each consecutive check for five hours, and neither filled their idiosomas. Similarly with D. hydei, two B. mali females attached to the tarsus after the 1st, 2nd, 3rd, 4th and 5th hours and 1 female, who during 4 consecutive hours of observation stuck once to the wing and another time to the tarsus, also remained flat.

Mite survival

In tests with D. melanogaster, only one female of B. mali died, out of 30 tested mites. It was attached to the cervix of the fly after the 1st and 2nd hour, and to the cervix of a dead fly after the 3rd, 4th and 5th hours of observations. In the test with D. hydei, 6 females of B. mali died, out of 30 examined individuals. Five dead female mites had not previously attacked the flies, and one had been attached exclusively to the fly's tarsus. We also noted two escapes of mites from the observational chamber through the vent. One female (which previously had not been observed to attack the fly) escaped from the chamber with a living D. hydei, after the 3rd hour (the mite’s absence was noted at the 4th hour), and another one after the 5th hour of its attachment to the cervix of a first live and then dead D. hydei (mite’s absence noted at the 24th hour).
